# Erratum for Exposure to Concentrated Ambient Fine Particulate Matter Induces Vascular Endothelial Dysfunction via miR-21

**DOI:** 10.7150/ijbs.69524

**Published:** 2022-01-21

**Authors:** Jianwei Dai, Wensheng Chen, Yuyin Lin, Shiwen Wang, Xiaolan Guo, Qian-Qian Zhang

**Affiliations:** 1 GMU-GIBH Joint School of Life Sciences, Guangzhou Medical University, Guangzhou 510182, China;; 2 The State Key Lab of Respiratory Disease, Guangzhou Institute of Respiratory Disease, The First Affiliated Hospital, Guangzhou Medical University, Guangzhou 510120, China;; 3 Vascular Biology Research Institute, School of Basic Course, Guangdong Pharmaceutical University, Guangzhou 510006, China.

In our paper 1, the TIMP3 western blot image in Figure 3F should be corrected as follow.

## Figures and Tables

**Figure 3:**
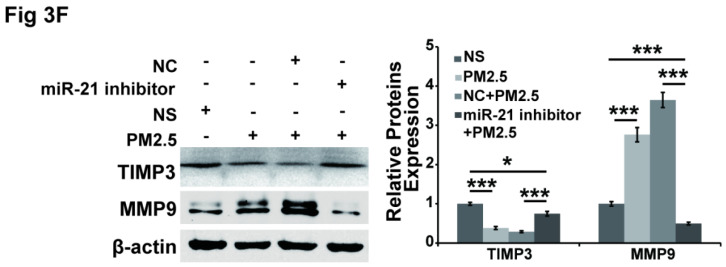
(F) Western blotting shows that the expression of TIMP3 was inhibited and MMP9 was up-regulated by PM2.5, and the effect was abolished by restraint of miR-21 in HUVECs. β-actin served as an internal control.*P< 0.05,**P< 0.01, ***P< 0.001.
